# Detrimental Synergistic Effects of Atmospheric Ozone and Polystyrene Nanoparticle Exposure on Human Adult Myogenic Progenitor Cells

**DOI:** 10.1155/bri/7493400

**Published:** 2025-11-14

**Authors:** Cristina Purcaro, Ester Sara Di Filippo, Cecilia Paolini, Piero Di Carlo, Stefania Fulle

**Affiliations:** ^1^Department of Neuroscience Imaging and Clinical Sciences, University of Chieti-Pescara, Chieti 66100, Italy; ^2^Interuniversity Institute of Myology (IIM), Perugia 06132, Italy; ^3^Center for Advanced Studies and Technology (CAST), University of Chieti-Pescara, Chieti 66100, Italy; ^4^Department of Innovative Technologies in Medicine & Dentistry, University of Chieti-Pescara, Chieti 66100, Italy

**Keywords:** atmospheric pollutants, human adult myogenic progenitor cells, ozone, polystyrene nanoparticles, skeletal muscle

## Abstract

Ozone (O_3_) and polystyrene nanoparticles (PNPs) display diffusive behavior that leads to toxicity in many tissues of the adult organism. In this study, we evaluated the interactions between atmospheric pollutants and human muscle, using human myogenic progenitor cells (huMPCs) derived from vastus lateralis skeletal muscle. To achieve this goal, O_3_ and PNPs were first tested individually to understand the impact of the single pollutant on huMPCs. Subsequently, pollutants were tested in combination to examine their potential synergistic effects, given the simultaneous presence of multiple pollutants in the atmosphere. Cell viability was assessed after treatment with O_3_ and PNPs, and it seems to be significantly affected in huMPCs exposed to the pollutants, tested both alone and in combination. Similarly, the differentiation capability of treated huMPCs was evaluated, and it was found to be significantly reduced compared to controls, especially when O_3_ and PNPs are tested in combination. Furthermore, an alteration in the expression of microRNAs involved in myogenic cells' proliferation and differentiation pathways was found. In light of the correlation between pollutants and increased oxidant levels, and O_3_'s ability to produce the superoxide anion, superoxide anion levels in huMPCs exposed to pollutants were also assessed, and an increase in this oxidant was recorded. Thus, this preliminary study suggests that exposure to O_3_ and PNPs affects human muscle, as it alters all the analyzed parameters in huMPCs, filling a gap in the current literature.

## 1. Introduction

Exposure to atmospheric pollutants is nowadays of significant concern [1, 2]. Natural events and human activities are both responsible for the worldwide spread of harmful chemicals. The contamination of air, soil, and water with hazardous compounds and toxic substances, leading to human organism adsorption, leads to health issues over time. The human body typically adsorbs environmental pollutants by inhalation and ingestion in different amounts. The potentially detrimental effect of this contact depends on the intensity and the duration of the exposure [3]. Once in the human body, many of these toxic compounds display diffusive behaviors, reaching even distant areas of the organism and interacting with target cells and tissues. In particular, the literature of the last decades described the interaction of O_3_ and polystyrene nanoparticles (PNPs) with the skeletal muscle system, by locally altering the muscular physiology [4, 5].

PNPs are nanoplastics of small dimensions (< 1 μm) that originate from anthropic activities and are released into the environment via industrial discharge but also wastewater and sewage systems. These nanoparticles can also originate from the fragmentation of bigger plastic debris caused by abrasion, biodegradation, or UV rays [6]. PNPs mainly enter the body by mouth or skin, but evidence of hair passive absorption has been found as well [7, 8]. Their toxicity is strictly linked to accumulation levels and nanoparticle size, causing oxidative stress, inflammation, immunotoxicity, and cytotoxicity [7]. In particular, nanoparticles cause disruption of mitochondrial stability with increased levels of reactive oxygen species (ROS) and release of proinflammatory and proapoptotic factors, eventually ending in cell death [9, 10]. For instance, Shengchen and his collaborators carefully described the effects of microplastics on muscular regeneration in mice, highlighting the skeletal muscle involvement in environmental pollutants-mediated toxicity [4, 11]. In this paper, they found oxidative stress in satellite cells and inflammation during regeneration as expected, and disruption of the equilibrium between myogenic and audiogenic differentiation. Thus, the regeneration of the muscle fibers was impaired [4].

Regarding O_3_, it is a highly reactive gas whose exposure can cause a plethora of harmful conditions [5]. Interestingly, O_3_ absorption can vary due to differences in airways size, with children and women displaying the higher levels of absorption [12]. O_3_ manages to spread in the human body after inhalation causing, for instance, oxidative stress and inflammation in the upper airways, depending on O_3_ concentration and length of exposure [13]. Such pollutant shows low solubility in water making it hard to be removed by the upper respiratory tract. Most of the inhaled O_3_ reaches the lower tract in which the epithelial lining fluid is present. Here, O_3_ dissolves reacting with the component of the epithelial lining fluid, which comprises a mixture of lipids, proteins, and antioxidant compounds. As soon as O_3_ reacts with them, reactive byproducts are produced which are responsible for the spreading of oxidative stress and inflammation in the respiratory tract and even to further tissues and organs, for instance, skeletal muscles [14, 15].

Interestingly, the ongoing toxicity of PNPs is aggravated when other pollutants are present. The co-exposure of pollutants can be responsible for greater damage to tissues and organs. Indeed, these nanoparticles can mediate the local adsorption of other pollutants, ending in a synergistic effect with higher toxicity [16, 17]. Thus, the aim of this study is to observe the influence of O_3_ and PNPs on human myogenic progenitor cells (huMPCs).

## 2. Materials and Methods

### 2.1. Human Subjects' Recruitment and Muscle Biopsy Sampling

Healthy male subjects (*n* = 18) between 20 and 80 years old were recruited to undergo muscle biopsies of *vastus lateralis* to obtain huMPCs. Before the study, all the subjects signed written informed consent and the Ethics Committee for Biomedical Research, University of Chieti (PROT 16/19 COET) ensured that the study adhered to the Declaration of Helsinki (as amended in 2000). Furthermore, the health status of all subjects was assessed before the biopsy to avoid the presence of exclusion criteria, such as pathologies linked with muscle, bone, or heart. ECG and blood pressure were also measured to guarantee they were within the standard ranges. The tiny percutaneous needle technique was applied to perform the muscle biopsies [18, 19]. Following the biopsy, muscle tissues were collected in a 10-mL tube with HAM's F-10 medium (#ECB7503SL, Euroclone, Italy) plus 50 mg/mL Gentamicin (#ECM0011D, Euroclone, Italy).

### 2.2. Culture of huMPCs

huMPCs were obtained from muscle biopsies following the procedure described by Fulle et al. [20]. Briefly, muscle biopsies were quickly defrosted at 37°C and were cut into small pieces with sterile scissors. Each piece was plated with a drop of FBS (FBS, #CHA1111L, Euroclone, Italy) and placed in the incubator at 37°C with 5% CO_2_. 48 h later, the growth medium (GM) was added in the muscle biopsies dishes [21].

After 7–14 days, the first mononucleated cells migrated out of explants. When cells reached 70%–80% of confluence, they were split. The population doubling level (pdl) was calculated after each split procedure, given that the first cells derived from biopsies have 1 pdl, using the following formula: (log 10(*Nt*/*Ni*)/ln 2), where *Nt* indicates the number of cells counted during the slip procedure and *Ni* indicates the number of cells seeded during the previous split procedure.

Cells were cultured in differentiation medium (DM) until complete differentiation into myotubes, which occurs after 7 days [21].

### 2.3. Characterization of huMPCs

To assess the myogenic purity and the effectiveness of differentiation of cell culture, an immunocytochemical assay for the detection of desmin and myosin heavy chain proteins was performed following the protocol described by Pietrangelo et al. [22]. Briefly, cells were incubated with one of the following primary antibodies: monoclonal mouse primary antibody anti-human desmin, clone D33 (#M0760, DAKO, USA), or monoclonal mouse primary antibody anti-myosin heavy chain, clone MF20 (Developmental Studies Hybridoma Bank, University of Iowa, USA). Myogenic cells and differentiated cells can be detected due to their brown staining (#K5001, DAKO, USA), with hematoxylin used for counterstaining.

Ten random pictures of each well were taken using a digital camera (Canon EOS 350D, Japan) connected to the microscope (Leica Microsystems CMS GmbH, Germany) at 20x magnification. Then, the number of cells positive for desmin antibody was quantified using the ImageJ software, and the percentage of myogenic purity was calculated as the ratio of the number of desmin-positive cells to the total number of cells multiplied by 100. Instead, the differentiation rate was calculated as fusion index percentage (FI%) as the ratio of the number of nuclei in the myotubes to the total number of nuclei multiplied by 100.

### 2.4. Treatment: Pollutants Exposure Protocols

huMPCs were treated with O_3_ and PNPs. Regarding O_3_, it was tested at a concentration of 120 ppb (i.e., around 240 μg/m^3^), which is the alert threshold: when a 1-h concentration exceeds this limit, the WHO guideline reports significant health effects, substantial proportion of vulnerable populations will be affected [23], and therefore, the EU legislation requires countries to take immediate action [23]. The O_3_ was directly injected into the incubator at the selected concentration using the device Ozone Calibration Source™—Model 306 (OCS™) from 2B Technologies Inc. (USA) connected to the incubator.

Concerning the PNPs, nanoparticles of different sizes (100, 200, 600, and 800 nm; #43302, #69057, #5067, and #65984, Sigma-Aldrich, USA) were purchased as 10% (w/v) solid suspensions. This corresponds to a stock concentration of approximately 100 mg/mL. For the experiments, the nanoparticles were diluted 1:1000 or 1:2000 in GM or DM, resulting in final concentrations of 100 and 50 μg/mL, respectively.

The two pollutants were tested firstly alone and then in combination.

huMPCs cultured in a standard incubator without any treatment were used as controls. Indeed, in this case, the incubator was not linked with the ozone device, and PNPs were not added to the huMPC medium.

### 2.5. Viability of huMPCs

huMPC viability was evaluated through a colorimetric assay that measured a blue formazan product reduced from tetrazolium-based compound by living cells (MTT assay). To perform the assay, huMPCs were seeded in 96-well plate and, after treatment, 20 μL of 3-(4,5-dimethyl-2-thiazolyl)-2,5-diphenyl-2H-tetrazolium bromide at a concentration of 5 mg/mL (#475989, Sigma-Aldrich, USA) was added to each well. Subsequently, huMPCs were incubated for 3 h at 37°C and then centrifuged at 2000 rpm for 15 min. The supernatant was removed, the formazan crystals at the bottom of the well were dissolved in 200 μL of dimethyl sulfoxide (DMSO, #D5879, Sigma-Aldrich, USA), and the plates were incubated for another 30 min at 37°C. Finally, a reading on the spectrophotometer Synergy H1 BioTek (Agilent, USA) was performed at a wavelength of 540 nm.

MTT assay was carried out after 24, 48, and 72 h of huMPC incubation with O_3_ and PNPs of different sizes (100, 200, 600, and 800 nm) at two different dilutions (1:1000, 1:2000) tested alone. Subsequently, MTT was performed at the same timepoints (24, 48, and 72 h) after exposing huMPCs to O_3_ in combination with 100 or 200 nm PNPs diluted 1:1000 and 1:2000.

### 2.6. Differentiation Capability

The differentiation capability of huMPCs was assessed through an immunocytochemistry assay using MF-20 antibody for the myosin heavy chain protein detection, as previously described in the huMPC characterization paragraph. huMPCs were cultured for 4 and 7 days in DM while being incubated with O_3_ and nanoparticles of different sizes (100 and 200 nm) diluted 1:1000 and 1:2000 either alone or in combination before performing the protocol for differentiation capability.

### 2.7. miRNA Expression

The total RNA isolation was performed on huMPC pellets after 24 h of treatment with O_3_ and PNPs of different sizes (100 and 200 nm) at 1:1000 dilution, firstly tested alone and then in combination. To achieve this goal, miRNeasy Micro Kit (#217084, Qiagen, Netherlands) was performed according to the manufacturer's protocol, and the total amount of RNA was quantified using the NanoPhotometer® NP80 (Implen, Germany). After RNA extraction, the reverse transcription of RNA into cDNA was carried out through the High-Capacity cDNA Reverse Transcription kit (#4368814, Applied Biosystems, USA). Finally, the qRT-PCR was performed in 96-well plates with the TaqMan probes and the specific TaqMan Universal Master Mix II, no UNG (#4440040, Applied Biosystems, USA) using the QuantStudio™ 7 Pro Real-Time PCR System (Thermo Fisher Scientific, USA). Specific miRNA sequence probes (Applied Biosystems, Life Technologies, Italy) were used to evaluate the miRNA expression: hsa-miR-1 (#002222), hsa-miR-133a (#002246), hsa-miR-133b (#002247), hsa-miR-206 (#000510), hsa-miR-23a (#000399), and hsa-miR-23b (#000400). The ubiquitously hsa-miR-16-5p (#000391, Applied Biosystems, USA) was used as an internal control. Furthermore, the relative quantification of miRNA targets was performed using the Δ*Ct* formula (*Ct*_miRNA⁣of⁣interest_–*Ct*_miR‐16_), following the *Ct* method.

### 2.8. Superoxide Anion Levels

Mitochondrial superoxide levels were assessed through the Mitochondrial Superoxide Detection Kit (Fluorometric, #ab219943, Abcam, UK), according to the manufacturer's instructions. huMPCs were seeded in a 96-well black-bottom plate and were treated for 24 h with O_3_ and PNPs of different sizes (100 and 200 nm) diluted 1:1000, tested both alone and in combination. After exposure to environmental pollutants, huMPCs were incubated with the fluorogenic reagent of the kit for 30 min at 37°C in the dark. Finally, the fluorescence was measured at an excitation wavelength of 540 nm and an emission wavelength of 590 nm using the spectrophotometer Synergy H1 BioTek.

### 2.9. Transmission Electron Microscopy (TEM)

The cells were washed twice in PBS at 37°C, fixed in 3.5% glutaraldehyde in 0.1 M sodium cacodylate buffer, pH 7.2, and then kept in fixative at 4°C before further use. For thin-sectioning, the cells were postfixed in 2% OsO4 for 2 h at room temperature and then contrasted in saturated uranyl acetate either for 2 h at room temperature. The samples were embedded in Epon 812. EM ultra-thin sections (∼50 nm of thickness) were cut from embedded samples using a Leica Ultracut R microtome (Leica Microsystem, Austria) with a 45° Diatome Ultra diamond knife (Diatome, Switzerland) and stained with lead citrate. Sections were viewed and photographed in a 120 kV JEM-1400 Flash TEM (Jeol Ltd, Japan) equipped with CMOS camera Matataki and TEM Center software (Jeol Ltd, Japan).

### 2.10. Statistical Analysis

The statistical analysis was performed through the Software GraphPad Prism, using Version 9.3.1 (GraphPad Software, USA). The data are reported as the mean ± SEM for the MTT assay and the superoxide assay kit, and mean ± SD for the differentiation assay and miRNA expression. Furthermore, the unpaired *t*-test was conducted in order to identify statistical differences, with a *p* value < 0.05 considered statistically significant.

## 3. Results

### 3.1. huMPC Viability

**Figure 1:**
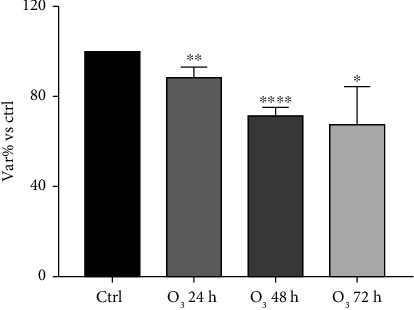
Viability of huMPCs exposed to O_3_. MTT assay of huMPCs: viability of huMPCs exposed to O_3_ (120 ppb) for 24, 48, and 72 h. Data are expressed as var% vs. ctrl. The means ± SEM of *n* = 40 were analyzed using unpaired Student's *t*-tests ^∗∗∗∗^*p* < 0.0001, ^∗∗^*p* < 0.005, and ^∗^*p* < 0.05 vs. ctrl.

**Figure 2:**
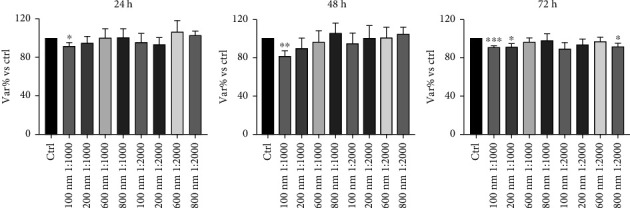
Viability of huMPCs exposed to PNPs. MTT assay of huMPCs: viability of huMPCs treated for 24, 48, and 72 h with nanoparticles of different sizes (100, 200, 600, and 800 nm) and dilutions (1:1000, 1:2000). Data are expressed as var% vs. ctrl. The means ± SEM of *n* = 40 were analyzed using unpaired Student's *t*-test. ^∗∗∗^*p* < 0.0005, ^∗∗^*p* < 0.005, and ^∗^*p* < 0.05 vs. ctrl.

**Figure 3:**
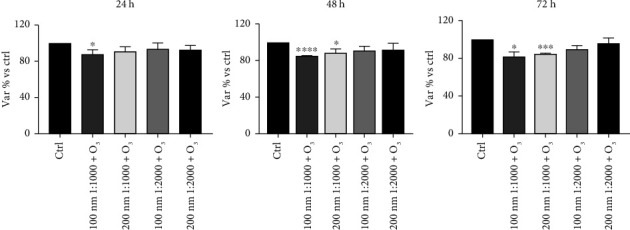
Viability of huMPCs exposed to O_3_ and nanoparticles. MTT assay of huMPCs: viability of huMPCs treated for 24, 48, and 72 h with nanoparticles of different sizes (100 and 200 nm) and dilutions (1:1000, 1:2000) upon O_3_ (120 ppb) exposure. Data are expressed as var% vs. ctrl. The means ± SEM of *n* = 40 were analyzed using unpaired Student's *t*-test ^∗∗∗∗^*p* < 0.0001, ^∗∗∗^*p* < 0.0005, and ^∗^*p* < 0.05 vs. ctrl.

Measurements of huMPC viability depicted the impact of the tested pollutants on these cells. Indeed, exposure to O_3_ tested alone for 24, 48, and 72 h showed a statistically significant reduction of viability compared to controls, already after 24 h of incubation (Figure 1). Similarly, huMPCs exposed for 24, 48, and 72 h to PNPs alone and dilutions showed a reduced viability, with a stronger effect related to smaller size and higher concentration of nanoparticles (Figure 2). Based on the results obtained, only the 100 and 200 nm nanoparticles were selected for the subsequent study and tested in combination with O_3_. Interestingly, huMPCs exposed for 24, 48, and 72 h to a combination of O_3_ and nanoparticles showed reduced viability in comparison to control cells, particularly when the dilution of nanoparticles was lower (Figure 3).

### 3.2. huMPC Differentiation

**Figure 4:**
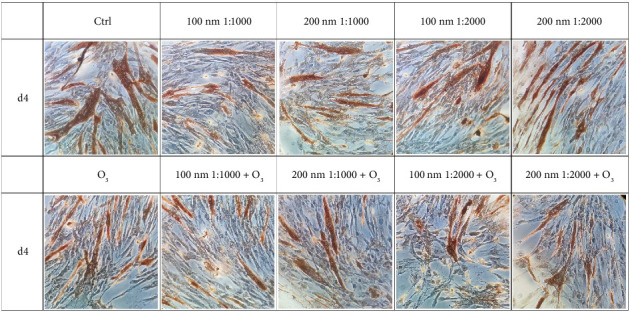
Immunocytochemistry on huMPCs with MF-20. huMPCs treated with O_3_ (120 ppb) and nanoparticles of different sizes (100 and 200 nm) and dilutions (1:1000, 1:2000), tested both alone and in combination. Day 4 (d4) of differentiation.

The exposure to the chosen environmental pollutants impacted on the differentiation of huMPCs as well. In detail, on Day 4, a general reduction in FI% was observed in huMPCs exposed to O_3_ and PNPs, both alone and in combination, compared to controls (Figure 4). Notably, the worst and statistically significant result was recorded after treatment with O_3_ in combination with 100 nm nanoparticles at 1:1000 dilution, as shown in Table 1.

**Figure 5:**
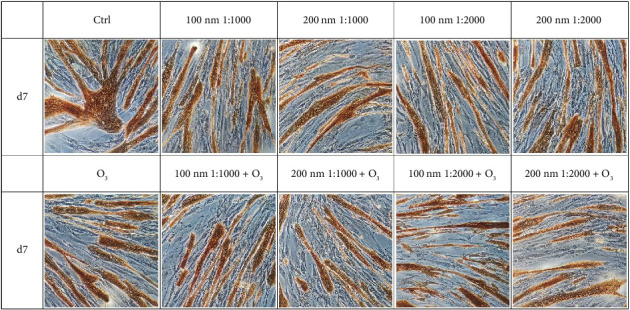
Immunocytochemistry on huMPCs with MF-20. huMPCs treated with O_3_ (120 ppb) and nanoparticles of different sizes (100 and 200 nm) and dilutions (1:1000, 1:2000), tested both alone and in combination. Day 7 (d7) of differentiation.

On Day 7, the FI% of huMPCs treated with both O_3_ and nanoparticles was significantly reduced when the pollutants were used alone or in combination (Figure 5). When combined, O_3_ and 100 nm nanoparticles at 1:1000 dilution demonstrated an even more detrimental effect, further reducing the FI% compared to pollutants tested alone (Table 2).

### 3.3. huMPC miRNA Expression

**Figure 6:**
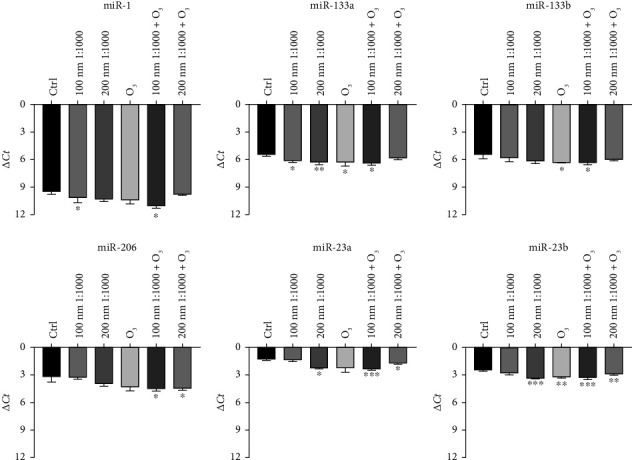
Modulation of miRNA expression (Δ*Ct*) levels in huMPCs upon exposure to environmental pollutants. qRT-PCR on huMPCs: miRNA expression treated with O_3_ at 120 ppb, nanoparticles (100 and 200 nm) diluted 1:1000, and the combination of nanoparticles and O_3_. Student's *t*-test. Data are shown as mean ± SD of *n* = 3. ^∗∗∗^*p* < 0.0005, ^∗∗^*p* < 0.005, and ^∗^*p* < 0.05 vs. ctrl.

When treated with O_3_ and PNPs, huMPCs displayed alteration in miRNA levels. In detail, Figure 6 highlights a general trend of downregulation of miRNA expression after 24 h of treatment with PNPs compared to control cells. Furthermore, 24 h of exposure to O_3_ was able to affect miRNAs' levels of treated cells compared to controls, leading also in this case to a general trend of downregulation. This trend is confirmed for the treatment with the combination of two pollutants too. Particularly, the results showed a statistically significant downregulation of all the miRNAs analyzed in huMPCs treated with 100 nm nanoparticles at 1:1000 dilution upon O_3_ exposure compared to controls.

### 3.4. huMPC Superoxide Anion Levels

**Figure 7:**
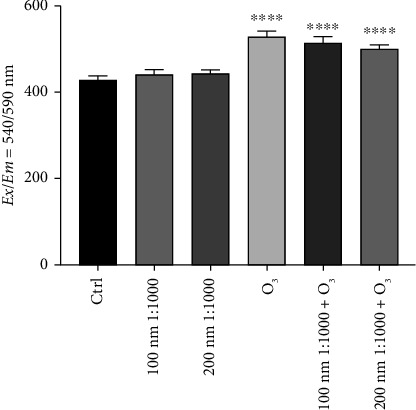
Levels of superoxide anion in huMPCs after 24 h of exposure to O_3_ and nanoparticles. Intracellular levels of superoxide anion of huMPCs treated for 24 h with O_3_ at 120 ppb and nanoparticles (100 and 200 nm) diluted 1:1000, tested alone and in combination. Student's *t*-test. Data are shown as mean ± SEM of *n* = 15. ^∗∗∗∗^*p* < 0.0001 vs. ctrl.

huMPCs treated for 24 h with O_3_ and polystyrene, tested both alone and in combination, showed a significant increment of superoxide anion levels only when incubated with O_3_ compared to controls. Indeed, O_3_ alone caused a stronger effect on huMPCs, whereas nanoparticles alone did not induce any detectable effect. Nonetheless, significantly higher levels of superoxide anion are detected in huMPCs after treatment with the combination of the two pollutants, as shown in Figure 7.

### 3.5. Detection of Nanoparticles With TEM in huMPCs

**Figure 8:**
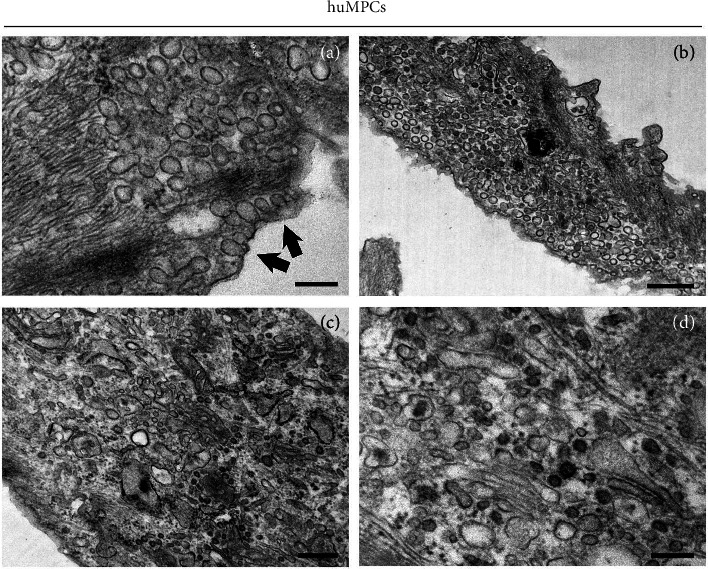
Cell membrane permeability to PNPs in huMPCs. Transmission electron microscopy analysis revealed movement of PNPs into membrane structures toward the cytoplasm. Active transport through the cell membrane (d) may represent a significant gateway into the cell after treatment with 100 nm PNPs. Bars: (a, b) 0.5 μm; (c, d) 0.2 μm.

TEM analysis of huMPCs treated with 100 nm PNPs provides detailed visual evidence of their uptake and localization within cellular structures. At the initial stage of internalization, PNPs can be observed near or at the cell surface, often interacting with the plasma membrane (Figure 8(a), arrows). PNPs are typically internalized through an active transport (phagocytosis), a process where the cell engulfs extracellular material by wrapping its membrane around the particle. The phagocytosis steps include formation of phagocytic cups around the nanoparticles, and inclusion of several subsequent nanoparticles inside the same cell and the process is complete when nanoparticles are encapsulated within a phagosome and transported inside the cell. Once internalized, nanoparticles appear dispersed within the cells, close to cellular compartments like the Golgi apparatus or endoplasmic reticulum (Figures 8(c) and 8(d)) or distributed randomly within the cytoplasm (Figure 8(b)).

## 4. Discussion

Considering the diffusive behavior of pollutants, in particular, PNPs and O_3_, with their known adverse effects on human health and in light of the absence of specific studies on human muscle, the aim of the study was to examine environmental pollutant interaction with huMPCs [24]. Indeed, given that adult muscle stem cells represent the main cell population involved in the regeneration of adult skeletal muscle, in this innovative study, we evaluated the direct effects of pollutants on skeletal muscle regeneration using a human myogenic stem cell model [25].

The obtained results highlight a negative impact of the pollutant on huMPC viability. In fact, Figures 1 and 2 are possible to observe a reduction of treated cells' viability compared to controls, which is significant already after 24 h of incubation with O_3_ or PNPs tested alone. Concerning O_3_, the concentration of 120 ppb was selected, as it represents twice the safe threshold for human health established by EU legislation, and it corresponds to levels repeatedly recorded in various regions of the US and Europe [26, 27]. Studies in literature demonstrate how 120 ppb O_3_ are able to exert toxic effects on several human cell types [26, 28]; however, data on muscle cells were lacking. Our findings demonstrate that the same harmful effect also occurs in huMPCs. Regarding PNPs, several studies in literature compare micro- and nanoparticle effects on cells reporting their size-dependent toxic impact. Indeed, smaller size particles are able to lead to the worst damage. This effect is linked with the significantly higher accumulation of smaller size particles, i.e., nanoparticles, founded in several organs in animal models [29, 30]. Our outcomes confirm the same effect also on human muscle: huMPCs treated with PNPs showed a significantly reduced viability compared to controls, in particular, when treated with the smallest nanoparticles (100 nm) diluted 1:1000 in all three time points considered. Instead, by using 600 and 800 nm nanoparticles, no relevant differences were found in relation to controls (Figure 2). Thus, it is possible to assume that there is a higher accumulation of 100 and 200 nm, compared to 600 and 800 nm, in organs such as muscles, causing more pronounced damage. In view of the evidence in the literature and the results obtained for cell viability, only 100 and 200 nm nanoparticles were selected for the subsequent studies. In fact, cell viability was evaluated after huMPC incubation with O_3_ in combination with the selected nanoparticles at different dilutions. The results also exhibited in this case a reduction in cell viability with the most significant effect linked to the treatment with the combination of O_3_ and nanoparticles at 1:1000 dilution. A significant result was already detected after 24 h for cells treated with O_3_ and 100 nm nanoparticles at 1:1000 dilution, as illustrated in Figure 3. Taken together, these results demonstrated how O_3_ and nanoparticles affected huMPC viability compared to controls, both alone and in combination.

Pollutants are also able to affect the differentiation capacity of huMPCs. Indeed, in the early differentiation timepoint, i.e., 4 days, the results reported in Figure 4 and Table 1 demonstrated a general trend of reduction in treated cells FI%, compared to controls. In particular, the significant and worst result was recorded for huMPCs incubated with O_3_ in combination with 100 nm nanoparticles used at 1:1000 dilution. Once fully differentiated, after 7 days, huMPCs showed a significant reduction of FI% for all the tested conditions evaluated compared to controls, as shown in Figure 5 and Table 2, with the worst effect still related to the combination of O_3_ with 100 nm nanoparticles used at 1:1000 dilution. Interestingly, the morphology of cells seems to be affected by pollutants as well. Indeed, treated huMPCs form smaller myotubes with a reduced number of nuclei compared to the control cells, as observed in Figures 4 and 5. These results demonstrate a negative effect of cited pollutants on huMPC differentiation capability, highlighting a possible synergistic effect of O_3_ and nanoparticles. Furthermore, in light of viability and differentiation capability outcomes linked to nanoparticle treatment of cells, the lower dilution was found to have a more damaging effect. Such data agree with the reported literature that underlines the higher toxicity of plastic particles when at higher concentrations [31]. For this reason, only the cited dilution was selected for the following studies. Considering, also, that the detrimental effects of pollutants were already evident after 24 h of treatment, only one time point was selected for the subsequent analysis, namely, 24 h.

Thus, in this innovative study, we demonstrated that pollutants impair the ability of huMPCs to repair and regenerate muscle tissue. To further evaluate the interaction of pollutants with huMPCs, the expression of miRNAs was analyzed. Indeed, myo-miRNAs, such as miR-1, miR-133a, miR-133b, and miR-206, are known to be associated with proliferation and myogenic differentiation pathways [32–34]. Their activity is pivotal for the proper muscle development as they have been found to be expressed at high levels during myoblast differentiation [35–37]. In addition, recent studies demonstrated the involvement of miR-23a and miR-23b in myogenic differentiation pathways [38]. The results in Figure 6 demonstrated a trend of downregulation for all miRNAs analyzed, using pollutants alone or in combination. The impact appears to be particularly pronounced in cells treated with O_3_ in association with 100 nm PNPs. Indeed, the downregulation effect after the cited treatment is significant for four of the six miRNAs analyzed (miR-133a, miR-206, miR-23a, and miR-23b). The obtained results confirm the findings of the previous immunocytochemistry assays (with MF20 antibody), which indicated a reduction in differentiation following treatment with pollutants and, particularly, with the combination of O_3_ and 100 nm nanoparticles diluted 1:1000. However, further research is needed to investigate the underlying mechanisms and signaling pathways through which the altered miRNAs may exert their effects.

Finally, levels of superoxide anion were investigated considering that pollutants can be responsible for oxidative stress, increasing ROS levels and causing oxidative damage to mitochondria [34, 39]. In particular, O_3_ is a powerful oxidant and most of its toxicity is mediated via reactive free radicals, especially superoxide anion, which O_3_ is able to generate as it rapidly decomposes in the aqueous phase [40–43]. The results, reported in Figure 7, exhibited an increase in superoxide anion levels after treatment with O_3_ alone compared to controls, while nanoparticles alone seemed not to affect such ROS levels at all. When O_3_ and nanoparticles are used in combination, superoxide anion levels in treated huMPCs are higher than in controls, although the effect seems to be related only to O_3_ action. Interestingly, these results suggest a mechanism of toxicity that is exclusively O_3_-dependent, driven by superoxide overproduction that is not affected by the co-presence of PNPs at all.

Taken together, all the results obtained demonstrate the detrimental effect of both O_3_ and PNPs on huMPCs, impairing their ability to repair and regenerate muscle tissue. These pollutants are able to affect cell viability and differentiation capability, also leading to a miRNA expression alteration and to an increase in superoxide anion levels. Regarding nanoparticles, the effect is related to the dimension and the dilution tested. In detail, the smallest size nanoparticles (100 nm) used at lower dilution (1:1000) showed the worst effect on all the parameters considered. The mechanism of action used by 100 nm nanoparticles to exert their toxic effect is probably related to their accumulation in the cytoplasm of huMPCs, as shown in Figure 8. Indeed, TEM analysis demonstrated that 100 nm nanoparticles are internalized by the cells, resulting in alterations in the physiology of huMPCs.

Moreover, the data suggest a synergistic detrimental effect of O_3_ and nanoparticles on huMPCs, since the most significant and worst effect is related to the combination of the two pollutants. Finally, the pollutant effects are detectable in the short-term since damage were recorded already after 24 h of incubation.

## 5. Conclusion

The results obtained in the present study demonstrate the detrimental effect of both O_3_ and PNPs on huMPCs, which are responsible for muscle repair and regeneration. In detail, O_3_ significantly reduced cell viability and differentiation capacity, and it led to a general downregulation of all the miRNA analyzed, in particular, miR-133a, miR-206, miR-23a, and miR-23b, which are associated with proliferation and myogenic differentiation pathways. Moreover, O_3_ alone induced a marked increase in superoxide anion levels, indicating an O_3_-dependent oxidative stress response, whereas nanoparticles alone did not significantly increase superoxide levels. On the other hand, PNPs also negatively affected cell viability, differentiation capacity, and miRNA expression, but did not significantly influence superoxide levels. The experiments conducted on huMPCs prove that smaller size nanoparticles, specifically 100 nm, are the most harmful due to their ability to be internalized by the cells, as shown by TEM analysis. The dilution is another parameter linked to PNPs impact: In fact, the worst effect was recorded after treatment with nanoparticles diluted 1:1000.

When tested in combination, O_3_ and PNPs produced the most pronounced detrimental outcomes on cell viability, differentiation, and miRNA regulation, suggesting a potential synergistic effect, while oxidative stress remained primarily driven by O_3_.

Together, these results offer a first insight into the negative impact of O_3_ and PNPs on human muscle, filling the gap in the current literature. Given the central role of huMPCs in skeletal muscle regeneration, our findings suggest that exposure to such pollutants may impair muscle homeostasis and contribute to conditions, such as reduced regenerative capacity, metabolic dysfunction, or sarcopenia. Our findings also underscore the broader impact of environmental pollutants and the urgent need to develop strategies to reduce human exposure. Indeed, the detrimental effects of pollutants may be much greater than commonly assumed and are not limited to the upper airways, but also extend to skeletal muscle and potentially other tissues.

In the future, it will be important to deepen the knowledge about the molecular mechanisms of action of these pollutants to better understand how O_3_ and PNPs exert their toxic effects and to translate these findings into preventive or therapeutic strategies.

## 6. Limitations of the Study

One of the limitations of the study is the use of a 2D model. It would be interesting evaluate the impact of such pollutants on more complex in human in vitro models, such as 3D models. Indeed, human 3D in vitro models can enable more in-depth studies to describe the molecular impacts of environmental pollutants on human biology. Another limitation of the present study is the focus on spherical nanoparticles. It would be interesting to investigate whether nanoparticles with different shapes can induce similar effects.

## Figures and Tables

**Table 1:** Fusion index percentage of huMPCs after exposure to O_3_ and nanoparticles at day 4.

Sample	Fusion index (%)	Unfused desmin + cells (%)
Ctrl	38.70 ± 16.72	38.43 ± 05.62
100 nm 1:1000	27.82 ± 14.09	71.09 ± 13.48^∗∗∗^
200 nm 1:1000	29.22 ± 15.92	74.30 ± 14.34^∗∗∗^
100 nm 1:2000	31.05 ± 18.79	49.69 ± 10.90^∗^
200 nm 1:2000	33.54 ± 14.52	53.88 ± 20.92
O_3_	30.21 ± 14.39	62.11 ± 26.01
100 nm 1:1000 + O_3_	24.62 ± 16.99^∗^	81.14 ± 13.87^∗∗∗∗^
200 nm 1:1000 + O_3_	28.66 ± 19.85	46.59 ± 26.20
100 nm 1:2000 + O_3_	27.75 ± 21.58	45.33 ± 08.47
200 nm 1:2000 + O_3_	28.06 ± 13.44	81.19 ± 08.86^∗∗∗∗^

*Note:* Immunocytochemistry on huMPCs with MF-20. Fusion index of huMPCs treated with O_3_ (120 ppb) and nanoparticles of different sizes (100 and 200 nm) and dilutions (1:1000, 1:2000), tested both alone and in combination. Day 4 of differentiation. Data are shown as percentage of fusion index (mean ± SD) of *n* = 15; percentage of unfused cells positive to desmin antibody (mean ± SD) of *n* = 15. Student's *t*-test.

^∗∗∗∗^ *p* < 0.0001, ^∗∗∗^*p* < 0.0005, and ^∗^*p* < 0.05 vs. ctrl.

**Table 2:** Fusion index percentage of huMPCs after exposure to O_3_ and nanoparticles at day 7.

Sample	Fusion index (%)	Unfused desmin + cells (%)
Ctrl	61.92 ± 11.16	11.29 ± 05.13
100 nm 1:1000	48.95 ± 13.38^∗∗^	25.77 ± 10.58^∗∗^
200 nm 1:1000	50.09 ± 13.09^∗∗^	19.71 ± 09.48^∗^
100 nm 1:2000	49.73 ± 11.87^∗∗^	21.43 ± 06.40^∗∗^
200 nm 1:2000	49.77 ± 13.15^∗∗^	27.24 ± 20.43
O_3_	50.26 ± 07.36^∗∗^	29.37 ± 12.19^∗∗^
100 nm 1:1000 + O_3_	45.82 ± 09.58^∗∗∗^	19.46 ± 06.73^∗^
200 nm 1:1000 + O_3_	46.51 ± 09.83^∗∗∗^	28.55 ± 10.19^∗∗∗^
100 nm 1:2000 + O_3_	48.66 ± 09.10^∗∗∗^	32.04 ± 12.67^∗∗∗^
200 nm 1:2000 + O_3_	47.01 ± 07.60^∗∗∗^	33.12 ± 12.36^∗∗∗^

*Note:* Immunocytochemistry on huMPCs with MF-20. Fusion index of huMPCs treated with O_3_ (120 ppb) and nanoparticles of different sizes (100 and 200 nm) and dilutions (1:1000, 1:2000), tested both alone and in combination. Day 7 of differentiation. Data are shown as percentage of fusion index (mean ± SD) of *n* = 15; percentage of unfused cells positive to desmin antibody (mean ± SD) of *n* = 15. Student's *t*-test.

^∗∗∗^ *p* < 0.0005, ^∗∗^*p* < 0.005, and ^∗^*p* < 0.05 vs. ctrl.

## Data Availability

Data will be made available upon request.
